# Socio-Emotional Wellbeing in Parents of Children with Neurodevelopmental Disorders: A Systematic Review

**DOI:** 10.3390/children13010099

**Published:** 2026-01-09

**Authors:** Mª Lourdes Álvarez-Fernández, Celestino Rodríguez

**Affiliations:** 1 Department of Psychology, Sociology and Philosophy, University of León, 24071 León, Spain; 2 Department of Psychology, University of Oviedo, 33003 Oviedo, Spain; rodriguezcelestino@uniovi.es

**Keywords:** family context, parental intervention, socio-emotional variables, wellbeing, neurodevelopmental disorders

## Abstract

**Background/Objectives:** Neurodevelopmental disorders (NDDs) require contextual approaches emphasizing family roles. Parents of children with NDDs face a complex socio-emotional reality. They may experience high levels of stress, fatigue, depression, and feelings of guilt and uncertainty, and they are often left feeling isolated and unsupported. All of these factors increase their socio-emotional vulnerability and affect their children’s wellbeing. A significant part of the available evidence has focused on parents of typically developing children or on a single construct. For these reasons, and considering the impact of the COVID-19 pandemic, the aim of this study was to review interventions targeting the improvement of the socio-emotional wellbeing of parents of children with NDDs, in order to characterise recent research, the specific constructs addressed, and the effectiveness of interventions. **Methods:** No prior protocol/registration. ERIC and Web of Science databases (selected for their broad multidisciplinary coverage in psychology and social sciences) were searched from 2020–2025 (last search: 7 September 2025), limited to English/Spanish publications. Inclusion criteria encompassed parents/primary family caregivers of children with NDDs receiving socio-emotional programs. Two independent reviewers screened the titles/abstracts and full texts, resolving disagreements through discussion. Following PRISMA 2020 guidelines, this systematic review employed narrative synthesis without risk-of-bias assessment and included 16 studies (approximately, 1100 participants). **Results:** The analysis indicated a scarce but growing scientific output, with a complex methodological landscape showing promising preliminary convergence in intervention outcomes. Interventions effects appeared mediated by cultural suitability, accessibility, and contextual alignment. **Conclusions:** Future work should pursue multisystemic approaches engaging diverse societal contexts and agents to optimize child and family wellbeing.

## 1. Introduction

Neurodevelopmental disorders (NDDs), which arise in early years, are characterised by the presence of alterations, deficits, or difficulties in physiological, neurological, and cognitive functioning or development, affecting personal, academic, and social contexts [1,2]. NDDs include autism spectrum disorder, attention deficit and hyperactivity disorder, specific learning disorders, communication disorders, motor disorders, intellectual disabilities, tic disorders, and other unspecified neurodevelopmental disorders [3]. Although prevalence varies between countries, due to a lack of uniform diagnostic criteria, approximately one in six children aged between 3 and 17 years old presents a neurodevelopmental disorder, which is about 17% of the child and adolescent population [4].

NDDs can be studied from two complementary perspectives. One is internal or personal, which focuses on the factors affecting the severity of the disorder, with an emphasis on personal and neuropsychological variables. The other is a contextual perspective, focusing on analysing the external variables that can affect the prognosis and severity along with the child’s future development [5,6,7]. This contextual approach includes research in NDD in the family environment. In the DSM, and in line with family systems theory [8], interactions between members of the family—between parents and between parents and children—are considered key processes that determine the outcome of children’s development as well as children’s and the family’s wellbeing. The nature and quality of family interactions are also influenced by family-level characteristics (such as parental stress) and by child-level characteristics (for example, the needs presented by the child).

Parents of children with NDD face a complex socio-emotional reality, which will vary according to the type of disorder and the specific needs related to it [1,5,9]. While parents do report positive benefits of raising a child with NDD, they have also been shown to have a high risk of suffering high levels of stress and emotional difficulties [10]. To a greater or lesser extent, parenting in these cases, associated with the demands of care and different types of difficulties the children have, seems to have a negative impact on parents, who often find themselves questioning their parenting abilities, feeling ambivalence, and worrying about their children’s futures [11]. Parents of children with NDD have higher rates of divorce, and higher levels of social isolation, stigmatisation, and care burden [12]. In such scenarios, one significant challenge for parents is finding sufficient time to deal with their own needs. Parents often do not have time for self-care, a lack that is associated with greater levels of stress, lower perceived social support, worse psychological wellbeing, and exhaustion [13,14,15,16]. All of these factors increase socio-emotional vulnerability in the family context, with direct, negative repercussions on children’s overall wellbeing [14,16,17].

The scientific literature in the field indicates that parents often feel misunderstood, unsupported, under-appreciated, and ill-prepared to support their children’s needs without additional help [18]. In this regard, provision of proactive support at the level of family systems is seen as critical and is increasingly recommended. Parents with effective resources and support can feel empowered to cope with and adapt to their situations, reducing the socio-emotional risks noted above, and strengthening the family as a whole, their children’s general wellbeing and therefore their overall health [19].

### The Present Study

In summary, it is essential that research also addresses NDD from a contextual perspective, considering the family context, in relation to the impact that it can have on parents, who should in turn be considered active agents in their children’s wellbeing. While the family context is essential to any child’s development, it is particularly important where there is NDD. Understanding the socio-emotional dimension of parenting in this context is key to comprehensively addressing both family functioning and child development.

In this regard, most interventions in the field of NDD have, nevertheless, continued to adopt an internal or personal approach, aiming their efforts towards personal and neuropsychological variables [16]. From the contextual perspective, a significant part of the available evidence has focused on parents of normotypically developing children or on specific constructs such as parental stress. As a result, the findings to date have been heterogeneous and often not easy to compare.

Added to all of this is the impact of the COVID-19 pandemic, which meant a considerable increase in, among other things, families’ levels of stress, anxiety, and emotional overload [20]. The suspension of in-person support services, confinement, and greater care demands led to increased parental vulnerability, highlighting the need to reinforce parents’ socio-emotional competencies even more [21,22]. This scenario also demonstrated the need for more flexible, accessible, sustainable interventions that incorporated virtual or hybrid formats, maintaining emotional support and guidance for families [21,23,24].

Because of the reasons outlined above, a systematic review is needed allowing the available evidence to be summarised and synthesised. Given the considerable social and emotional impact of the post-COVID period, this review deliberately will focus on studies published between 2020 and 2025 that examined programmes targeting socio-emotional reinforcement aimed at parents, or main family caregivers, of children presenting NDD. This timeframe makes it possible to capture recent research developed in response to new challenges, such as increased parental stress, service disruptions, and the expansion of virtual or hybrid intervention formats. It aims to give an up-to-date picture of interventions in the field, mapping the latest scientific output. In addition, it will look at how participants are characterised (parental predominance, presence of other carers, and types of NDD dealt with, among others), the methodological designs used, the main socio-emotional dimensions examined, and the interventions with the greatest reported benefits.

The resulting synthesis will be a basis for optimising the design of more comprehensive programmes for parents in the future. This will help produce more coherent, evidence-based programmes that effectively respond to families’ socio-educational needs and consequently to the overall wellbeing of children with NDD.

## 2. Method

The literature review followed the criteria in the PRISMA statement [25], with the aim of systematically identifying and examining relevant studies. No prior protocol/registration was established. Following PRISMA 2020 guidelines, this systematic review employed narrative synthesis without risk-of-bias assessment and included 16 studies.

The systematic review included studies that (a) analysed interventions, or the results of interventions, aimed at promoting parents’ (or main family carers’) socio-emotional variables; (b) had samples of parents or carers whose charges were children or adolescents, under 18 years old; (c) were published in English or Spanish in the ERIC or Web of Science databases; and (d) were published in the previous five years, between 2020 and 2025.

**Figure 1:**
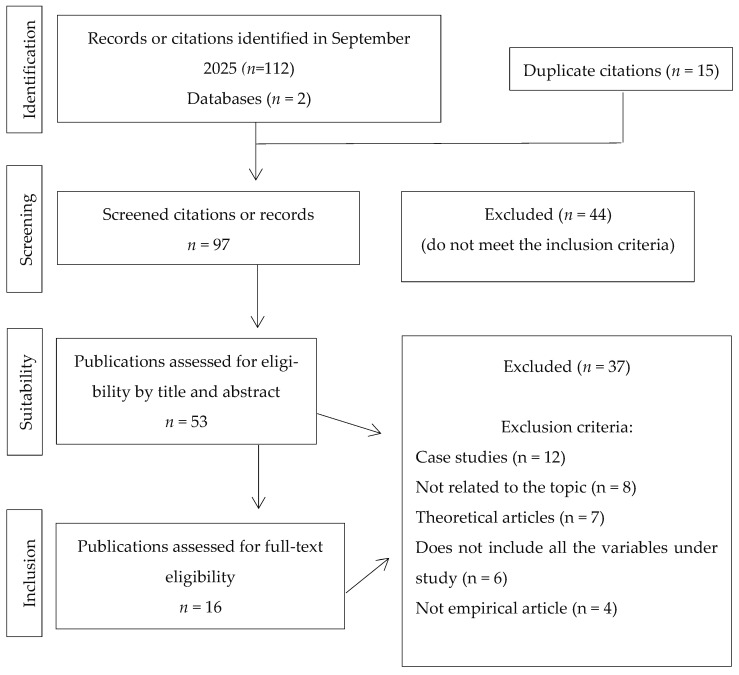
PRISMA Flow diagram of the systematic review. Adapted from [25].

Figure 1 summarises the process for selecting the sample of articles, along with the results, including the reasons for exclusion during the full review of the text. The bibliographic search was performed during the first week of September 2025 in Web of Science and ERIC, as noted above (last search: 7 September 2025). They were selected for their broad multidisciplinary coverage in psychology and social sciences. In Web of Science, the search was also refined by indexed journals, selecting Science Citation Index Expanded (SCI-Expanded) and Social Sciences Citation Index (SSCI). The search used the following Boolean terms in the title, abstract, or key words: (“neurodevelopmental disorders” OR “special educational needs”) AND (“parent” OR “caregiver” OR “family”) AND (“socioemotional” OR “parental well-being”) AND (“intervention” OR “program” OR “support”).

In the first stage, all of the possible results were considered, and duplicates between databases and searches were removed. In the second stage, the titles and abstracts were assessed to refine the list, excluding articles that did not refer to the topic of the review or which did not meet the previously established criteria. Lastly, the full texts of the remaining articles were reviewed to produce the final sample.

Two independent reviewers screened the titles/abstracts and full texts. Disagreements were resolved through discussion. No snowballing was applied, as the ERIC and Web of Science databases provided 112 relevant records within the restricted timeframe (2020–2025).

### 2.1. Inclusion and Exclusion Criteria

The criteria for including articles were: (a) that the articles were published between 2020 and 2025. The 2020–2025 timeframe was chosen to focus on recent evidence reflecting post-COVID challenges, providing an updated synthesis amid limited prior reviews on this specific scope; (b) that the samples were parents or, alternatively, other primary family caregivers of children with NDD, and that the children were minors; (c) that they were studies staging interventions focused on promoting parents’/caregivers’ socio-emotional variables; and (d) that they were published in English or Spanish.

Studies were excluded if they met the following criteria: (a) studies published as book chapters or conference summaries rather than scientific articles; (b) case studies; and (c) meta-analyses or reviews.

### 2.2. Data Collection

Following the PRISMA criteria, using the two aforementioned databases in the identification phase, a total of 112 articles were produced by the search terms and publication filters (2020 to 2025). Each of those articles was reviewed against the inclusion and exclusion criteria, with 15 duplicates removed before the screening phase, leaving a total of 97 pre-selected articles.

In the screening phase, 44 of the 97 pre-selected articles did not meet the inclusion criteria, as they did not deal with the required variables or were not scientific articles. This left 53 articles for suitability review.

Reviewing the suitability indicated that 37 articles did not meet the inclusion criteria for various reasons: they were cases studies, were not specifically related to the topic, were theoretical articles or meta-analyses, did not include all the variables being studied, or the variables were not clear.

Finally, 16 articles were selected that met all of the criteria, which were subsequently analysed.

## 3. Results

### 3.1. Year of Publication, Language, Journal, and Country

Since 2020, based on the review, research on the topic has been scarce. From 2020 to 2022 only a single article was published each year. 2025 saw the most articles, a total of 6, published. All of the articles were published in English in 8 different journals. Only the Journal of Applied Research in Intellectual Disabilities and the Journal of Autism and Developmental Disorders published more than one article on the topic during the last five years. The largest proportion of the studies (31.25%) were conducted in the United States (Table 1).

### 3.2. Variables Related to Participants in the Studies Analysed

There was variety in both sample composition and sample size. Mothers predominated, although some studies included fathers or other family members considered to be principal caregivers, reflecting a broader approach to the family context. Sample sizes ranged from small, with 10 participants, to a larger sample of 261 parent–child dyads. Parents’ age was only specified in five studies, and the mean age was 39.9. Lastly, in 4 of the studies (25%), the participants came from challenging contexts or were at psychosocial risk.

In relation to the types of NDD the studies addressed, 9 of the interventions considered a variety of NDDs (56.25%), while 6 focused specifically on the autism spectrum (37.5%) and 1 on intellectual disability (6.25%). The age ranges of the children were also very diverse, from studies that looked at broad age ranges (e.g., from 0 to 17 years old) to others that examined narrower ranges (e.g., from 2 to 4 years old). The predominant age range was around 3 to 11 years old, with the largest proportion of studies dealing with parents of preschool or primary school children (Table 2).

### 3.3. Methodological Characteristics of the Studies Analysed

All of the studies demonstrated a common interest in improving parents’ or main family carers’ socio-emotional wellbeing. However, each study addressed specific aspects with complementary approaches. 10 of the studies (62.5%) focused specifically on evaluating aspects referring to preliminary efficacy, feasibility, acceptability, and implementation processes of the interventions, some of which were innovative such as online programmes or those based on acceptance and commitment therapies. Other objectives ranged from exploring and describing carers’ subjective experiences to identifying risk and protective factors associated with parental stress, or analysing the relationships between variables such as self-compassion, self-efficacy, empowerment, and quality of family life. Consequently, the methodological designs were varied, from quasi-experimental pre–post studies without control groups to controlled randomised trials with parallel groups and pre, post, and follow-up evaluations.

In terms of evaluation instruments, the studies used a wide variety, combining quantitative methods—which included standardised scales and validated questionnaires—and qualitative methods—predominantly semi structured interviews or focus groups, along with emotional journals and self-reports. The most commonly measured variables were parental stress, in 9 studies (56.25%), depression in 5 studies (31.25%), and parental self-efficacy and anxiety, in 4 studies each (25%). The studies measuring aspects such as feasibility and acceptability of interventions did so mainly via checklists, registers of attendance, and ad hoc satisfaction questionnaires.

Finally, it is worth noting that 4 interventions (25%) were designed with the additional consideration of being applied in very specific situations of psychosocial risk, such as in populations with cultural or linguistic barriers to accessing services, or in rural communities with limited economic resources.

All of the information noted above related to the focus or objective of each study, the participants, methodological design, measures, and evaluation instruments are classified and specified in the table below (Table 2).

A high methodological heterogeneity was observed, which was addressed through a thematic narrative synthesis. This approach made it possible to identify convergences in the effectiveness of the interventions, moderated by factors such as cultural adequacy and format accessibility (Table 2, Table 3 and Table 4).

### 3.4. Description of the Interventions in the Reviewed Studies

Firstly, looking at the format of the interventions, 10 were implemented in groups (62.5%), 3 were individual (18.75%), and 3 were mixed modality (18.75%). In terms of how they were delivered, 7 were in-person (43.75%), 4 were online or virtual (25%), and 2 were in a hybrid format (12.5%). In addition, 1 intervention that was initially designed to be delivered in person was adapted to an online format due to the COVID-19 lockdown, and another intervention could be applied in both formats.

Two of the interventions (both online) were self-directed (12.5%), 7 were facilitated by specialists in mental health, psychology, or associated areas (43.75%), and the remained were co-facilitated by specialists and parents or family carers (43.75%).

The interventions lasted between 2 and 18 sessions, with the most common duration being 6 to 10 sessions. Sessions lasted from 30 min for individual sessions to up to 3.5 h in intensive group sessions. The session content, related to direct care for parents/main family carers, focused on a variety of topics: parental self-care, stress management, parenting skills, self-compassion, parental empowerment, reduction in stigma, and aspects related to physical wellbeing. From a methodological perspective, the interventions used cognitive-behavioural therapy, acceptance and commitment therapy, psychoeducation, experiential interventions, mindfulness, and parent-mediated behavioural strategies.

Specific information about the main characteristics of each intervention reviewed is provided in detail in the table below (Table 3).

### 3.5. Principal Results and Findings from the Analysed Studies

The table below (Table 4) summarises the aspects that the interventions were identified as working on, related to parental wellbeing at the socio-emotional level, along with the main related results. It also shows a combination of specific numeric data and qualitative data in relation to percentages of effectiveness and adherence to the interventions.

There was a convergence in terms of efficacy, with most interventions reporting, for example, significant reductions in parental stress and parents’ emotional health, along with increased active participation and social networks from the participants. In addition, there was over 70% adherence reported, which reinforces the idea that these interventions are viable and accepted.

Interventions such as [4,26,37] stand out for applying techniques centred on self-compassion, mindfulness, and based on Acceptance and Commitment Therapy (ACT), achieving consistent effects in improving parents’ wellbeing and excellent adherence (between 80–85%). On the other hand, programmes such as [27,38,39]—which combined professional facilitation, active participation from trained parents and community promotors, in online and in-person formats—demonstrated outstanding results in self-efficacy, emotional regulation, and social support, with high scores in satisfaction and adherence above 70%. The interventions focusing on training behavioural skills and social communication, such as [32,33], stand out for their positive effects on behavioural management and improvements in family communication, again with good adherence (>75%).

The evidence indicates that the effectiveness of the interventions in this review is mediated by cultural suitability, the format, and the relevance to the specific needs of each family context. Because of that, interventions incorporating a multidimensional, adaptive focus achieve better overall results. Parental self-efficacy was strengthened through empowerment programs and Acceptance and Commitment Therapy interventions, which fostered more effective coping strategies. Social support acted as a key mediator in reducing stress and feelings of isolation, with better outcomes observed in group-based or parent co-facilitated interventions. Improvements in parental self-efficacy stemmed from specific skill training and peer emotional validation, which significantly reduced perceived burden. Social support, particularly in group-based programs, emerged as a key resilience mechanism, moderated by contextual factors such as service accessibility and cultural barriers.

## 4. Discussion

This systematic review looked at studies published between 2020 and September 2025 on interventions focused on improving or promoting socio-emotional wellbeing in parents (or main family carers) of children with NDD. The review confirms that, although scientific output has been scarce, it is growing, particularly from 2023 onwards. This has perhaps been largely driven by the negative impact of the COVID-19 pandemic on these families, because lockdowns and suspension of support services led to them facing an increased care load and more stress.

The participants in the interventions were largely mothers. This is another confirmation of the fact that mothers tend to take on the bulk of primary care in the family context, with greater levels of stress according to the literature in the field [10,11]. This situation underscores why mothers have been the main focus of the interventions’ attention. The review also showed the wide range of sample sizes and greater attention to autism spectrum disorder and intellectual disability. The main ages of children covered by the interventions was between 3 and 11 years old, which is perhaps justified by the high prevalence of these disorders and their early diagnostic visibility [3].

The methodological designs and aspects of the interventions analysed in this review also reflect a wide diversity, from quasi-experimental pre-post designs without control groups, to randomised controlled trials with parallel groups, combining quantitative, qualitative, and mixed approaches. On the one hand, this picture shows the growing maturity of this line of research and the richness and complexity of the field. On the other hand, it reflects the rapid changes and urgent adaptations forced by the COVID-19 pandemic [31]. That said, it is worth noting that a significant majority of the studies used quasi-experimental pre–post designs without control groups, with a quantitative approach, prioritising the evaluation of feasibility and the preliminary effectiveness of the interventions.

Turning to the characteristics of the interventions themselves, in-person, group formats, facilitated by specialists, or co-facilitated by trained parents, predominated over self-directed formats. Nonetheless, there was evidence of an increased tendency towards online or hybrid formats, again driven by the impact of COVID-19. Both formats, online and hybrid, have been shown to be viable, increasing the flexibility available to interventions, and facilitating participation in rural environments or places with limited resources, although challenges remain such as those related to accessibility and technological and social obstacles, which need to be addressed to ensure equity [34].

The number of sessions and how long they last varied according to the formats and modalities, the types of facilitation and the methodological approach of the interventions. With regard to the latter, the approaches mainly combined psychoeducation, cognitive-behavioural therapy, acceptance and commitment therapy, experiential intervention, mindfulness, and parent-mediated behavioural strategies. They addressed topics within socioemotional wellbeing, linked to parental self-care, stress management, parenting competencies, communication and social skills, managing challenging behaviours, self-compassion, parental empowerment, reduction in stigma, and aspects of physical wellbeing. The most common methodological approach was psychoeducation to address parental self-care and stress, the socioemotional variables that were most commonly present in the interventions in this review.

On this point, it is worth noting that the review found promising preliminary convergence across the 16 interventions analysed, despite their methodological diversity, with significant reductions in parental stress, improvements in emotional health, and greater active participation or improvements in parents’ social networks. More specifically, the interventions based on acceptance and commitment therapy, mindfulness, and self-compassion stood out for their consistency. These types of experiential techniques may be more accessible than others for families in challenging situations [4,26,37]. Co-facilitated interventions exhibited high percentages of satisfaction [27,38,39], and those that included parent-mediated behavioural strategies or that addressed family communication achieved high percentages of adherence. In this regard, the scientific evidence about the benefits of peer-directed self-help groups is sound. The support they provide has been shown to be essential in reducing feelings of isolation, stigmatisation, and internalisation of self-stigma [20,27]. Creating safe spaces for exchanging experiences and mutual validation promotes feelings of belonging, normalisation, and empowerment [30,33].

In short, the evidence suggests promising preliminary effectiveness of these interventions, but this effectiveness is also mediated by cultural suitability, accessibility, and whether they are aimed at specific contextual needs [34,35,36,39]. In low-resource settings, short asynchronous online programs [4,32] proved most feasible. Peer co-facilitation should be prioritized when professional access is limited [27,30].

## 5. Conclusions

Interventions aimed at the parents or family carers of children with NDD should be considered an area of particular interest, owing to the socio-emotional challenges these families face [20,30]. The present systematic review has allowed us to take a detailed snapshot of the current scientific picture in the field, and shows that these types of interventions are key tools in helping families face that challenge, indirectly contributing to the children’s wellbeing and quality of life [14]. That said, there are some considerations that should be borne in mind that may offer greater robustness and soundness to this field of research, related for example, to more robust methodological designs, longitudinal follow-up of interventions to consolidate knowledge of their impact and sustainability, and the balance between scientific validity and practical applicability, among other aspects.

The findings of our systematic review have notable implications, especially given the scarcity of research over recent years, although it is growing. This scarcity makes it even more important to analyse the socio-emotional impact on parents, mostly mothers, who face high levels of stress, anxiety and depression, overload, and social isolation, aggravated by the COVID-19 pandemic, which underscores the urgency of family context approaches [21]. For this reason, various practical implications at the family, educational, political, and social level are discussed below.

Firstly, at the family level, we should bear in mind that raising a child with NDD produces socio-emotional maladjustment in parents that may lead to less adaptive parenting styles, increasing the burden of care and stigmatisation. Consequently, it is essential to develop accessible, culturally appropriate family programmes that equip parents with skills for self-care, stress management, and empowerment [40]. In this regard, interventions should be multidimensional, as well as directly addressing child wellbeing.

Secondly, on an educational level, parents’ socio-emotional state is also a priority. Children with NDD present difficulties and vulnerabilities that affect school and family contexts. This is why education professionals must be trained in socio-emotional competencies to help prevent parental exhaustion and to promote group interventions. Seeking family involvement is essential, and it needs to be quality involvement, through two-way communication between school and the home [19].

On a political level, the most important implication is related to the promotion of inclusive policies in line with socio-emotional care in all of the environments surrounding children with NDD and their parents, considering their day-to-day challenges. It would be useful to design early detection programmes and access to services that combine educational, social, family, and mental health work, encouraging co-facilitation by skilled, trained parents to reduce stigma and social isolation and to optimise community resources, with particular attention to vulnerable family situations [41]. These interventions must be within comprehensive systems of family care in order to maximise their impact on quality of life and social inclusion [30,34,42,43].

Finally, in the mental health sphere, there should be better collaboration with schools to offer integrated support to students with NDD and their families through specific programmes [44]. In addition, parents and educators need good quality training in early detection and the application of evidence-based interventions [5,45,46,47,48].

Despite these practical implications, the review does have a series of limitations that need to be considered when interpreting the results. In the first place, the sample of articles included for the review was small, as there were only a limited number of studies published on the topic in the time period selected, and the search was limited to only two databases. On the other hand, no protocol/registration was established prior to the search nor was a risk-of-bias assessment conducted. In addition, the selected studies also included interventions that were not limited solely to developing or strengthening socio-emotional variables, to a single modality, format, or specific intervention characteristics, cultural contexts, family scenarios, participants or NDD, or specific age ranges, which may limit how representative and generalisable the findings are. These are aspects that future research may address specifically in a variety of ways, with the present systematic review being seen as a first approach. Finally, in accordance with the implications noted above, it would be interesting for future studies to address the same research objective, but from a multisystemic perspective. Consequently, the present review emphasises the need for future interventions to address parental socio-emotional wellbeing with the involvement of different contexts and agents in society, with the same ultimate aim of improving the overall wellbeing of children with NDD.

## Figures and Tables

**Table 1:** Bibliometric properties of the articles included in the systematic review (N = 16).

Descriptive Variable	Fr	%
Year of publication		
2020	1	6.25
2021	1	6.25
2022	1	6.25
2023	4	25.0
2024	3	18.75
2025	6	37.50
Language		
English	16	100.0
Journal		
Advances in Neurodevelopmental Disorders	1	6.25
Autism	1	6.25
Autism Research	1	6.25
Child: Care, Health and Development	1	6.25
Journal of Applied Research in Intellectual Disabilities	5	31.25
Journal of Autism and Developmental Disorders	5	31.25
Journal of Intellectual & Developmental Disability	1	6.25
Research in Developmental Disabilities	1	6.25
Sample Country		
Canada	2	12.50
USA	5	31.25
United Kingdom	4	25.0
India	1	6.25
Italy	1	6.25
South Africa	1	6.25
Sweden	1	6.25
Turkey	1	6.25
Total	16	100.00

**Table 2:** Focus and methodological characteristics of each study.

Authors (Year)	Objective	Participants	Methodological Design	Measures (Instruments)
Ahmed et al. (2023) [4]	Evaluate the feasibility of a short, asynchronous, online intervention focused on promoting self-compassion.	50 parents (48 mothers, 2 fathers; mean age: 42.1 years), with children aged between 3 and 17 years old with: Down syndrome, ASD, ADHD, intellectual disability, overall developmental delay, others (spina bifida, cerebral palsy, apraxia, oppositional defiant disorder).	Pre-post design, without control group.	- Self-compassion (SCS scale). - Overall wellbeing (WEN-WBS). - Depression and stress (DASS-21). - Feasibility (Bowen’s model: 5 dimensions).
Bergman et al. (2023) [26]	Evaluate the feasibility and preliminary results of a group intervention based on Acceptance and Commitment Therapy.	94 parents (85 mothers, 9 fathers) of children with various disorders (ASD, autism spectrum disorder, ADHD, among others), with children aged 2 to 17 years old.	Pre, post, and follow up design, without control group.	- Parental stress (PSS). - Anxiety and depression (HADS). - Experiential avoidance in parenting (PAAQ). - Mindfulness (MAAS). - Strengths and difficulties in the children (SDQ). - Satisfaction, credibility, and usefulness of the intervention (CEQ; SEF & PEF).
Boule et al. (2023) [27]	Examine the emotional experience during a group support program for wellbeing.	104 parents (mainly mothers: 92 versus 8 fathers) of children up to 7 years old with intellectual disability and developmental difficulties.	Qualitative longitudinal study with repeated measures based on emotion journals and self-reports.	Emotional experience (online emotion journal).
Buyuk & Ozmen (2025) [28]	Evaluate the effectiveness of an empowerment programme based on reducing stress, improving self-efficacy and family empowerment.	69 mothers of children with ASD aged 6 to 14 years old: - Experimental group: 34. - Control group: 35.	Randomised controlled trial with two non-blinded parallel groups. - Experimental group: 4 training sessions and 2 individual motivational interviews. - Control group: standard practice. Pre and post design	- Parental self-efficacy (PSES). - Carer overload (ZCBS). - Perceived stress (PSS-14). - Family empowerment (FES).
Fante et al. (2025) [29]	Explore the impact of ASD on quality of life, and how a multidisciplinary intervention with parental participation influences quality of life and the process of adaptation.	31 parents (16 mothers and 15 fathers: 42.55 years old), with children diagnosed with level 2 or 3 ASD severity, aged 5 to 11 years old.	Qualitative transversal study based on semi-structured interviews, with inductive (bottom up) thematic analysis without a control group.	Quality of life: semi structured interview about quality of life, parents’ experiences, perception of the intervention, difficulties, and available resources.
Gore et al. (2022) [30]	Explore experiences after attending a group intervention programme, as well as the group processes and mechanisms from the carers’ perspectives.	35 family carers (90% mothers; mean age 36.9 and 38.8 years old) of children aged 2 to 4 years old presenting overall developmental delay, Down syndrome, ASD, or other genetic conditions.	Qualitative study based on individual interviews and focus groups about the experience held after the intervention.	Interviews and focus groups about: - Emotional wellbeing and resilience. - Feelings of belonging and group social support. - Parental self-efficacy. - Coping with stress. - Self-care. - Strategies to deal with challenging behaviours and improve family emotional balance.
Ibrahim et al. (2025) [31]	Analyse changes in mental health, mindful parenting, and parenting practice after participation in a community cognitive-behavioural group therapy programme.	77 parent–child dyads (mean age: 42.47 years old): verbally capable autistic children aged 8–13 years old.	Quasi-experimental pre-post study, without a control group.	- Stress and anxiety (DASS-21). - Mindful parenting (BMPS). - Parenting practices and family adjustment (PAFAS).
Ingersoll et al. (2024) [32]	Compare effectiveness of 2 parent-mediated distance intervention models (self-guided and with therapeutic support) in fidelity to the intervention, wellbeing, and engagement with the programme.	64 parents (mean age: 35.27 years old) of autistic children aged 1.5 to 8 years old.	Randomised controlled trial with 3 parallel groups. Pre and post evaluation and follow up.	- Parental self-efficacy (PSOC). - Knowledge about the intervention. - Perceived positive impact (FIQ). - Parental stress (PSI-SF). - Acceptability and satisfaction (STP). - Perceived obstacles (BTPS). - Technological fluency (CEW). - Expectations (CEP-Q).
Lodder et al. (2020) [20]	Evaluate feasibility, acceptability, and initial impact of a programme aimed at improving mental health and how it helps in protecting against stigmatisation.	17 parents of children with ASD up to 10 years old: - Experimental group: 9. - Control group: 8.	Randomised controlled trial with mixed quantitative and qualitative measures. Pre, post, and follow-up evaluations.	- Mental health (MHI-5). - Stigma (PCSS). - Self-esteem (Rosenberg’s scale). - Self-compassion (SCS-SF). - Positive meaning in the role of carer, self-blame, perceived social support (MOS). - Social isolation (UCLA). - Feasibility (retention and attendance rates, Fidelity of implementation, opinions about randomisation). - Acceptability (participation in focus groups, satisfaction comments, quantitative indicators).
Ondrušková et al. (2023) [33]	Evaluate implementation process of a group intervention.	261 parent–child dyads (children aged 2.5 to 5 years old presenting moderate to severe intellectual disability, with challenging or problematic behaviours): - Experimental group: 155. - Control group: 106.	Evaluation of process within a multi-centre randomised controlled trial.	- Fidelity (checklist adapted from the i-Basis Intervention Fidelity Rating Scale). - Dose and reach (attendance data). - Adaptations (systematic record of adaptations). - Acceptability (semi-structured individual topic interviews and parental satisfaction questionnaire).
Proctor et al. (2024) [34]	Evaluate the impact of a group intervention for reinforcing skills, focused on empowerment, peer support, social inclusion, exchange of knowledge and defensive skills.	37 parents who participated in 17 parent groups, with children under 10 who had developmental difficulties. A rural setting in the south of India.	Qualitative study based on focus groups held 6 months after the intervention, analysed via hybrid thematic analysis (deductive and inductive).	- Experiences of peer support, social inclusion, knowledge exchange, and exchange of defensive skills (discussion guide in focus groups).
Safer-Lichtenstein et al. (2023) [35]	Evaluate feasibility and acceptability of 2 parental interventions.	60 carers (mainly mothers) of children aged 0 to 5 years old with developmental delays. Split into two intervention groups: - Group 1: 30. - Group 2: 30. Spanish speakers residing in the United States.	Randomised controlled trial with a mixed approach. Pre, post, and follow-up evaluations.	- Parental satisfaction (PSQ). - Parental stress (PS-4-SF). - Childish behaviour (CBCL). - Acculturation (VIA). - Session attendance. -Assessment of the experience, challenges, value (focus groups).
Schelbusch et al. (2024) [36]	Evaluate feasibility, acceptability, and possible impact of a short wellbeing programme based on acceptance and commitment therapy.	10 carers (9 mothers and 1 grandmother; Mean age: 41.27 years old) of children with developmental difficulties aged between 4 and 11 years old. Participants were residents in a rural community with limited resources in South Africa.	Quasi-experimental pilot study. Pre and post evaluation without a control group.	- Psychological Flexibility (AAQ-II). - Depression (PHQ-9). - Anxiety (GAD-7). - Perceived social support (MSPSS). - Positive and negative impact of the disability on the family (FICD). - Parental wellbeing (McConkey). - Feasibility and acceptability (ad hoc feedback and post-session satisfaction form).
Schwartzman et al. (2021) [37]	Evaluate the efficacy of an intervention to increase resilience and reduce parental stress, and explore collateral effects on family, marriage, and child functioning.	35 parents of children with ASD aged between 4 and 11 years old.	Randomised controlled trial with two groups. Pre, post, and follow-up evaluations.	- Resilience (CD-RISC-25). - Stress, anxiety, and depression (DASS-21). - Parental stress (PSI-4-SF). - Mindful attention (MAAS). - Life orientation (LOT-R). - Acceptance and action (AAQ-II). - Self-compassion (SCS). - Family empowerment (FES). - Marriage quality (QMI). - Strengths and difficulties (SDQ). - Social responsiveness (SRS-2). - Disruptive behaviour (ABC-2).
Sutherland et al. (2025) [38]	Examine the experiences of participants and facilitators in a systematic family support programme, and the processes involved in the perceived changes.	8 parents (mainly mothers) and 9 family facilitators trained for the programme. Children aged 8 to 13 years old with ASD or intellectual disability.	Qualitative study evaluating processes and experiences, based on semi-structured interviews and focus groups, with thematic analysis applied to the participants’ and facilitators’ experiences and perspectives after the intervention.	- Semi-structured interviews.
Yu et al. (2025) [39]	Evaluate feasibility, acceptability, and preliminary effects of family intervention aimed at promoting health and wellbeing.	30 parents (mostly mothers) of children with intellectual disability and developmental difficulties, aged 6 to 17 years old. Hispanic families, with cultural and linguistic barriers to accessing services, in the United States.	Pilot study of intervention with a mixed approach. Pre-post evaluation.	- Self-efficacy in health (Self-Rated Abilities for Health Practices). - Social support (MSPSS). - Dietary behaviour (NCI Dietary Screener). - Physical activity (CHAMPS and PAQ-C). - Quality of life (PROMIS). - Index of unhealthy eating, screen time, depression, and stress (CESD-10, PSS-10). - Body mass index (BMI).

Note. ASD = Autism Spectrum Disorder; ADHD: Attention Deficit/Hyperactivity Disorder.

**Table 3:** Summary of the main characteristics of each intervention.

Authors (Year)	Intervention Characteristics
Ahmed et al. (2023) [4]	Short online intervention, 4 weeks, asynchronous (allowing access to the programme in line with participants’ other day to day commitments). Weekly modules lasting about 12 min (weekly reminders to finish modules): (1) Psychoeducation in self-compassion, (2) self-kindness, (3) shared humanity, and (4) mindfulness. Each module includes short psychoeducation about the topic being dealt with and a written experiential activity adapted to reflect parental experiences. Various activities: reflection exercises, writing about painful parenting events, and practices to promote self-compassion, self-kindness, recognition of shared humanity, and mindfulness.
Bergman et al. (2023) [26]	Group coping intervention based on Acceptance and Commitment Therapy (ACT Navigator) 5 group sessions each lasting 3.5 h and 1 reinforcement session lasting 2.5 h: (1) Where am I? Parenting a child with a disability; psychoeducation about parental stress, grief, the importance of rest; coping strategies; acceptance as an alternative to control and avoidance. (2) What is important for me? Impact of language on suffering; healthy distance from internal experiences; mindfulness in everyday tasks, play, and activity; valuable areas of life; accepting the child; self-compassion. (3) What can hold me back? Working on values: “Navigator for parenting and life”; identifying and managing internal and external barriers; unwritten rules that act as barriers; triggers in challenging parenting situations. (4) What do I need to do? The observer’s perspective and changing perspective; mindfulness and experiential acceptance in difficult situations; committed action that matters. (5) What do I promise myself? Creating an energetic balance: changing or accepting daily activities; engaging with parenting values; self-care; mindfulness in pleasant situations; self-compassion; psychologically flexible parenting. Closed groups of between 8 and 16 parents. Actively experiential methods: exercises, metaphor, dramatization, imaginary presentations, psychoeducation, mindfulness
Boule et al. (2023) [27]	Early Positive Approaches to Support (E-PAtS). Group intervention lasting 8 weeks involving psychoeducational strategies and emotional support, with weekly 2–2.5 h group sessions in-person or online: (1) how to deal with available support and services, (2) parental self-care, (3) sleep, (4) communication, (5) adaptive behaviours, (6 & 7) challenging behaviours, and (8) close and reflection. Intervention co-implemented by a mental-health specialist and a trained parent. Use of emotion journals to record and reflect on parents’ own emotions before and after each session.
Buyuk & Ozmen (2025) [28]	Parental empowerment programme in two parts: (1) training in empowerment for parents (4 in-person group sessions lasting 45 min each), and (2) motivational interviews (2 individual sessions). The topics addressed include the importance of play and communication, problems and managing them in nutrition, sleep, safety, self-care, and strategies for coping with parental stress. The sessions used interactive methods: structured presentations, guided discussion, and question and answer sessions. The motivational interview was performed by a certified researcher to ensure adherence and competence.
Fante et al. (2025) [29]	Mainly clinical intervention, but with activity in the home agreed with the family, based on TEACCH. Involving specialists from different areas (clinical psychologists, paediatricians, child neuropsychologists, speech therapists) in constant collaboration with the families. Variable duration, with individual, weekly 2–4 h sessions (the number of sessions is not specified: ongoing intervention). Parental training with an emphasis on active parental participation in all stages of diagnosis and rehabilitation, active collaboration between parents and specialists, parental training, psychological support, and generalisation of learning in the home. Topics: personalised TEACCH strategies, behavioural management, developing skills, parental and family support, training and support, social inclusion.
Gore et al. (2022) [30]	Early Positive Approaches to Support (E-PAtS). Group intervention, co-facilitated by a specialist and a family carer to offer early support to families. 8 weekly sessions lasting about two and a half hours, with groups of between 4 and 8 families. Combines practical exercises, group discussions, and psychoeducation. Subjects covered: access to services and training in assertiveness skills; carer’s wellbeing, promoting self-care and emotion management; helping the child sleep; communication and interaction with the child; development of adaptive skills; managing challenging behaviour (2 session); and incorporating knowledge and planning for the future.
Ibrahim et al. (2025) [31]	Community intervention based on cognitive behavioural therapy (CBT), with a strong parental participation component in 2 different directions: intervention for children and intervention for parents. The intervention for parents offers 3 possible formats: (1) 9 weekly forty-five-minute sessions, (2) 18 thirty-minute sessions, or (3) 2 two-hour sessions spaced each three weeks for 9 weeks. Regardless of format, the program begins with a 2-h informative session. Online version is available (applied during the COVID-19 pandemic).
Ingersoll et al. (2024) [32]	ImPACT Online: parent-mediated intervention programme, based on the Naturalistic–Developmental–Behavioural Intervention (NDBI). 2 possible formats: (1) Self-directed (over 6 months, 12 online interactive readings each lasting 75 min, depending on the pace of each participant). The readings cover: benefits of parent-mediated intervention, social communication, preparing the home for success, and other strategies (concentrating on the child, tailoring communication, creating opportunities, teaching new habits, and modelling interaction). (2) Self-directed + remote assistance from a therapist (includes 24 thirty-minute remote coaching sessions over 6 months: 2 sessions per week provided by a clinical specialist).
Lodder et al. (2020) [20]	Psychosocial intervention protecting against stigma (SOLACE programme). Eight weeks of weekly sessions (hybrid format: in-person sessions (1, 4, and 8) and online (2, 3, 5, 6, and 7)). The programme is based on psychoeducational strategies, cognitive restructuring, and techniques centred on compassion for reducing internalisation of stigma and protecting parents’ mental health. It includes group activities, discussion, and shared experiences to encourage identification with other parents and social support. Topics covered: myths and stereotypes about autism, managing stigma, positive meaning in care, resilience, self-esteem, social support, self-care and acceptance, as well as strategies for dealing with automatic negative thoughts related to stigma.
Ondrušková et al. (2023) [33]	Stepping Stones Triple P (SSTP) programme, parent-mediated. A psychoeducational programme based on the social learning model, comprising 6 in-person group sessions lasting 2.5 h and 3 individual thirty-minute calls over 9 weeks, given by trained therapists. Teaches behavioural strategies and management strategies to improve parents’ confidence and reduce challenging behaviours, promoting a positive parent–child relationship.
Proctor et al. (2024) [34]	Parental intervention programme with a group approach for reinforcing parenting skills. 8 group sessions lasting 2 h each. Uses a participative methodology where the facilitators guide, but the parents share and direct. Focus: parent empowerment and self-efficacy; peer support, connection between families facing similar challenges; social inclusion; sharing knowledge about resources, rights, and wellbeing related to disabilities; defensive skills and being advocates of children with disabilities; others such as health and community integration.
Safer-Lichtenstein et al. (2023) [35]	2 parental interventions (initially in-person but due to COVID-19, an online format was adopted): - Psychoeducation, stress reduction and mindfulness (MBSR). - Behavioural training for parents (BPT). 10 group support sessions, each lasting about 90 min, weekly over 10 weeks. Psychoeducation content + parental behavioural training or + mindfulness-based stress reduction techniques (MBSR).
Schelbusch et al. (2024) [36]	The Well-Beans for Caregivers programme (an adaptation of a wellbeing module for carers from the WHO Caregiver Skills Training (CST) programme). 3 in-person group sessions each lasting 2 h, over 3 weeks: - Session 1: introduction to acceptance and commitment, with emphasis on identifying personal values and the importance of accepting difficult emotions; activities to learn to be present and observe thoughts without judgement. - Session 2: techniques for coping with stressful thoughts and feelings related to caring for the child; exercises to improve psychological flexibility and manage emotional stress. Session 3: reinforcing commitment with actions in line with identified values; strategies for self-care, and construction of social support networks that strengthen resilience. The intervention is facilitated by a tiered model of training and supervision that includes mental health specialists and non-specialists. It is based on Acceptance and Commitment Theory (ACT). The sessions include stories, live exercises, and group discussions to help carers manage stress and improve their mental wellbeing. Strategies are applied to facilitate understanding, accessibility, and cultural adaptation, such as simplified language, use of illustrations, and the possibility of mixing local idioms with English.
Schwartzman et al. (2021) [37]	AMOR programme (Acceptance, Mindfulness, Optimism, Resilience Method). Facilitated by specialists in psychology 8 weekly group sessions lasting about 90 min each, which combine teaching, group discussion, behavioural practice, and weekly homework. Meditation exercises, reviewing homework, teaching new resilience strategies based on cognitive-behavioural therapy, full attention, self-compassion, and optimistic thinking. Content: managing stress, changing mentality about stress, gratitude, mindfulness, acceptance, facing grief and loss, optimism (in two sessions), self-compassion, and review to prioritise resilience.
Sutherland et al. (2025) [38]	Positive Family Connections Programme. Facilitated by trained family carers, supervised by psychologists. 6 online group sessions lasting 2 h with groups of 6–8 families (up to 2 carers per family). Topics covered: strengthening family relationships and wellbeing through peer support and promotion of healthy family processes.
Yu et al. (2025) [39]	Family PODER: an intervention culturally adapted for Latin American families. 10 weekly virtual individual sessions with a trained promotor, each lasting about 1 h, plus 3 group sessions reinforcing the content, two in-person and one virtual as an adaptation to overcome logistical obstacles. Individual session content: wellbeing and managing stress; balanced nutrition and food preparation; social support and healthy decision-making. The group sessions involve interactive activities to reinforce the learning, including practical demonstrations.

**Table 4:** Socio-emotional variables addressed, principal related results, and percentages of effectiveness and adherence to the interventions.

Intervention	Aspects Addressed to Improve Socio-Emotional Wellbeing	Main Related Results	Effectiveness (%)	Adherence (%)
Ahmed et al. (2023) [4]	- Self-compassion - Emotional wellbeing	- Reduced parental stress - Increased self-compassion	~60–65%	>80%
Bergman et al. (2023) [26]	- Parental stress - Self-care	- Improved emotional acceptance - Reduced parental stress	>70%	80–85%
Boule et al. (2023) [27]	- Parental self-care - Communication	- Improved self-care - Improved family life	65–75%	>75%
Buyuk & Ozmen (2025) [28]	- Stress management - Communication - Empowerments	- Increased parental self-efficacy - Reduced stress	70%	70–75%
Fante et al. (2025) [29]	- Behavioural management - Social abilities	- Reduction in disruptive behaviours - Improved social skills	>70%	75–80%
Gore et al. (2022) [30]	- Self-care - Adaptive skills	- Improved access to services - Promoted self-care - Improved adaptive skills	65–70%	>70%
Ibrahim et al. (2025) [31]	- Stress - Parenting skills	- Improved parenting abilities - Improved stress management	>70%	70–80%
Ingersoll et al. (2024) [32]	- Social communication - Behavioural management	- Improved social communication - Reduction in disruptive behaviours	75–85%	80%
Lodder et al. (2020) [20]	- Resilience - Stigma - Social support	- Increased resilience - Reduced stigma - Improved social support	65–70%	70%
Ondrušková et al. (2023) [33]	- Behavioural management - Parenting strategies	- Improved parenting strategies - Improved confidence	70–80%	>75%
Proctor et al. (2024) [34]	- Empowerment - Peer support	- Strengthened active participation - Strengthened social networks - Benefits in defensive skills	65–70%	75%
Safer-Lichtenstein et al. (2023) [35]	- Stress - Emotional wellbeing	- Reduced parental stress	>70%	70–75%
Schelbusch et al. (2024) [36]	- Self-care - Resilience	- Increased resilience - Reduced anxiety	70%	80%
Schwartzman et al. (2021) [37]	- Social support - Family wellbeing	- Strengthened social support - Strengthened family wellbeing	65–70%	75%
Sutherland et al. (2025) [38]	- Coping - Resilience	- Improved emotional wellbeing	70–80%	80–85%
Yu et al. (2025) [39]	- Stress - Emotional wellbeing - Parent empowerment - Social support	- Improved stress management	70–75%	70–75%

## Data Availability

The original contributions presented in this study are included in the article. Further inquiries can be directed to the corresponding author.
